# Ultra-Fast Microwave Synthesis of ZnO Nanorods on Cellulose Substrates for UV Sensor Applications

**DOI:** 10.3390/ma10111308

**Published:** 2017-11-15

**Authors:** Ana Pimentel, Ana Samouco, Daniela Nunes, Andreia Araújo, Rodrigo Martins, Elvira Fortunato

**Affiliations:** i3N/CENIMAT, Department of Materials Science, Faculty of Sciences and Technology, Universidade NOVA de Lisboa, Campus de Caparica, 2829-516 Caparica, Portugal; a.samouco@campus.fct.unl.pt (A.S.); daniela.gomes@fct.unl.pt (D.N.); andreiajoiaraujo@hotmail.com (A.A.); rm@uninova.pt (R.M.)

**Keywords:** ZnO, nanorod substrates, microwave irradiation, UV sensors

## Abstract

In the present work, tracing and Whatman papers were used as substrates to grow zinc oxide (ZnO) nanostructures. Cellulose-based substrates are cost-efficient, highly sensitive and environmentally friendly. ZnO nanostructures with hexagonal structure were synthesized by hydrothermal under microwave irradiation using an ultrafast approach, that is, a fixed synthesis time of 10 min. The effect of synthesis temperature on ZnO nanostructures was investigated from 70 to 130 °C. An Ultra Violet (UV)/Ozone treatment directly to the ZnO seed layer prior to microwave assisted synthesis revealed expressive differences regarding formation of the ZnO nanostructures. Structural characterization of the microwave synthesized materials was carried out by scanning electron microscopy (SEM) and X-ray diffraction (XRD). The optical characterization has also been performed. The time resolved photocurrent of the devices in response to the UV turn on/off was investigated and it has been observed that the ZnO nanorod arrays grown on Whatman paper substrate present a responsivity 3 times superior than the ones grown on tracing paper. By using ZnO nanorods, the surface area-to-volume ratio will increase and will improve the sensor sensibility, making these types of materials good candidates for low cost and disposable UV sensors. The sensors were exposed to bending tests, proving their high stability, flexibility and adaptability to different surfaces.

## 1. Introduction

In the recent years, a huge effort has been made to produce materials that can be used in different applications, such as nanoelectronics, optoelectronics, photonics, gas sensors, solar cells, photocatalysis, lab-on-paper for rapid diagnostic tests and antibacterial applications using flexible, biodegradable and green substrates, like cellulosic fiber-based substrates. The use of cellulosic substrates in these types of applications bring some advantages as cellulose is the Earth major biopolymer being suitable for low-cost applications, besides being flexible, lightweight, biocompatible and biodegradable [1,2].

Regular paper is composed by cylindrical cellulosic fibers, with diameters ranging from 20 to 50 µm and lengths that can reach 2 to 5 mm [3]. The larger roughness and porosity of the surface are intrinsic barriers to the development of electronic devices on the surface of this type of substrates. Nevertheless, the development of some devices on cellulosic fibers based substrates have already been reported [1,4,5,6,7,8,9,10].

Zinc Oxide (ZnO) is an *n*-type semiconductor with a wide and direct band gap of about 3.37 eV and a large free exciton binding energy of 60 meV at room temperature which allows it to act as an efficient semiconductor material [11]. ZnO possesses unique electrical, optical, photocatalytic and antibacterial properties, also being a low-cost material with a high surface reactivity. The physical and chemical properties of ZnO nanomaterials vary as a function of size, shape, morphology and crystalline structures.

Many efforts have been devoted to the development of different ZnO nanostructures with improved properties. The shape and the aspect ratio of this nanostructures are key factors that greatly influence the electrical and optical properties of ZnO material. Different techniques, precursors and solvents can be used to prepare a vast variety of nanostructures. Thus, new green synthesis strategies are vital for the development of novel nanomaterials [6,12].

Hydrothermal synthesis is a simple method that allows the production of uniform and well distributed ZnO nanorod arrays, and is generally associated with conventional heating, which is known to be inefficient, besides being time and energy consuming. Microwave irradiation, on other hand is a low cost technology due to its unique features such as short reaction time thus energy saving, enhanced reaction selectivity, homogeneous volumetric heating and high reaction rate [13]. In fact, microwave synthesis has been successfully employed for several sources of nanomaterials, shortening the synthesis reaction time [6,8,14,15,16,17,18].

As it is largely known, to synthesize a continuous ZnO layer using the hydrothermal synthesis method, it is imperative to use a seed layer [19,20]. In this sense, the use of different techniques for surface treatments have been reported by some authors to improve the surface wettability and adhesion. Examples of techniques employed include plasma treatment [21,22], wet chemical [23] and UV/Ozone treatment [24,25]. The UV/Ozone treatment is a simple, inexpensive and low-temperature method that allows simultaneously the removal of some surface contaminants and the polarization of ZnO seed layer surface.

The direct growth of ZnO nanorods on paper substrates has so far been reported by very few authors [20,26], however to the best of the author’s knowledge, this is the first time that ZnO nanorod arrays grown on different sources of paper substrates under microwave irradiation, with short synthesis time (10 min) and having UV/Ozone treatments inflicted to the seed layer have been reported.

Zinc oxide is frequently employed in a wide range of applications, from optoelectronics to biological fields [20,27,28,29,30,31]. Nevertheless, one of the most investigated areas for ZnO is UV/Ozone sensing, mainly due to its capacity of absorbing UV light, being transparent to visible light, presenting a high sensitivity and selectivity [32,33,34]. UV sensors are mostly used in UV source and environmental monitoring, space technologies, as well as in chemical and biological detection [35]. The use of ZnO nanostructures instead of thin films allows an increase in sensor responsivity and sensitivity due to a higher aspect ratio of length to diameter and higher surface area of nanostructures [36].

Many authors have reported the direct growth of ZnO nanorods to act as the photoactive layer in UV sensors on rigid (glass and silicon) [35,37] and flexible substrates (polymeric, textile and cellulosic substrates [26,36,38]). These reports showed responsivities ranging from 15 to 24 mA/W for glass and silicon substrates, while 0.022 µA/W was reported for other types of flexible substrates [35,36,37]. Nowadays, the scientific community seeks inexpensive, adaptable and flexible devices, which makes paper an appealing option for the next generation UV sensors devices.

The present work reports the synthesis of ZnO nanorods on tracing and Whatman papers by the hydrothermal synthesis method under microwave irradiation. The influence of synthesis temperature with a fixed time and the influence of UV/Ozone treatment on the ZnO seed layer have been studied. After an extensive structural, morphological and optical characterization of the synthesized nanorod arrays, the materials were tested as UV sensors.

## 2. Results and Discussion

### 2.1. Characterization of Paper Substrates: SEM, Thermal Analysis, XRD

As previously mentioned, the fact of cellulosic paper substrates present high roughness and porosity can be disadvantageous to the development of electronic devices. Nevertheless, two different types of substrates were chosen to investigate these effects, tracing paper and Whatman paper.

Prior to the seed layer deposition and growth of ZnO nanorod arrays, both pristine papers were fully characterized to understand their physical characteristics and limits (smoothness, impurities and temperature degradation).

**Figure 1:**
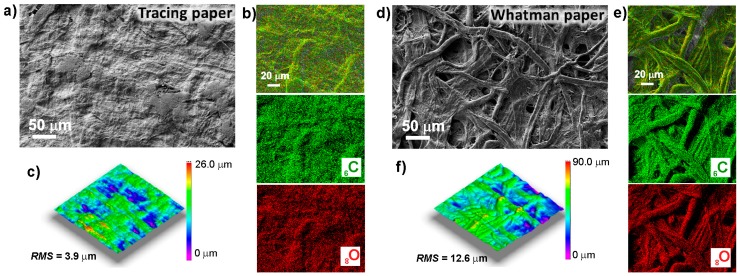
Scanning electron microscopy (SEM) images of (**a**) tracing paper and (**d**) Whatman paper substrates; (**b**,**e**) SEM images (artificial colored) together with the corresponding X-ray maps of C and O species of tracing and Whatman papers respectively; (**c**,**f**) Surface 3D profilometry of tracing and Whatman paper, respectively.

Figure 1 shows the scanning electron microscopy (SEM) images and the 3D profilometry measurements showing the roughness profiles of tracing and Whatman paper substrates. In the case of the tracing paper, it is almost impossible to distinguish the cellulose fibers. The Whatman paper revealed a high-density structure of intertwined cellulose fibers with a cylindrical and flat shape in the micrometer range. Thus, it is possible to observe that the tracing paper substrate presents a smoother surface when compared to the Whatman paper. This assumption is confirmed by the surface 3D profilometry measurements (see Figure 1c,f), in which it is possible to observe that tracing paper presents a root mean square (RMS) of 3.9 µm, while Whatman paper has a rougher surface with a RMS value of 12.6 µm. The energy dispersive spectrometry (EDS) images (see Figure 1b,e) confirm the absence of calcium carbonate (CaCO_3_) or any other contaminants.

**Figure 2:**
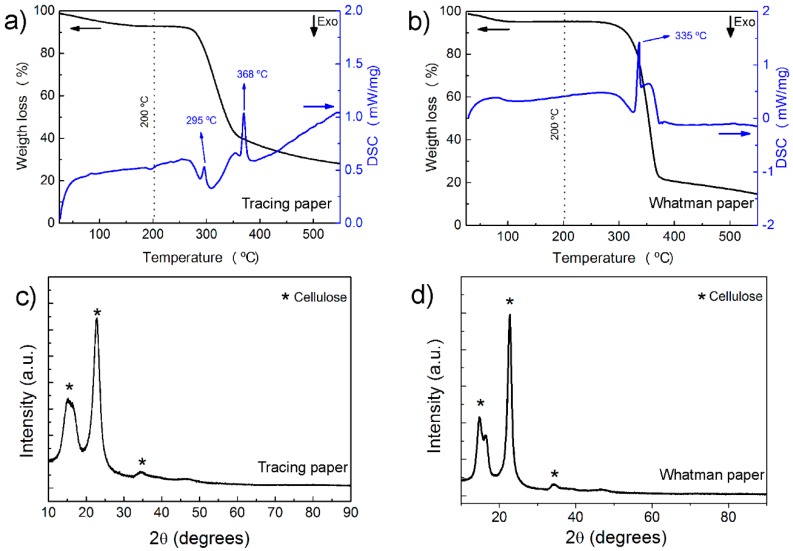
(**a**,**b**) Differential Scanning Calorimetry (DSC) and (**c**,**d**) X-ray diffraction (XRD) diffractograms of tracing and Whatman paper substrates, respectively.

To infer the maximum working temperature of tracing and Whatman paper substrates, differential scanning calorimetric (DSC) and thermogravimetry (TG) measurements were carried out. The results are presented on Figure 2a,b, for tracing and Whatman papers, respectively. It is well known that cellulosic fibers undergo for a rapid thermal degradation at low/moderate temperatures (below 400 °C). The thermal decomposition of cellulosic fibers consists on the degradation of several components that are on its composition—decomposition of hemicelluloses, pyrolysis of lignin, depolymerization of cellulose, active flaming combustion and finally char oxidation [39]. So, as mentioned by other authors [39,40,41], the decomposition of a cellulosic substrate can be divided in four steps. The first step occurs at temperatures between 40 and 120 °C and is related with the extraction of water or moistures presented on paper. The second step, usually is accompanied with a major mass loss, and can be correlated with the main degradation reaction of cellulose fibers due to depolymerization and carbonization of glycosyl units (being characterized by an endothermic peak approximately at 350 °C). The third step corresponds to the oxidation of the char produced with the fibers decomposition (this stage can be absent on some types of papers), occurring between 400 and 500 °C. The fourth stage usually occurs at temperatures above 630 °C and corresponds to the decomposition of calcium carbonate. The thermal decomposition of cellulosic fibers is greatly influenced by their structure and chemical composition. So as the chemical structure of the cellulosic fibers are arranged differently, they will decompose at different temperatures ranges and possess different decomposition profiles [39,41].

The DSC curve of tracing paper presents a small endothermic peak at 85 °C, accompanied by a small weight loss (about 6.40%), which corresponded to desorption or water evaporation from cellulose fibers. Between 280 and 400 °C an enhanced weight loss of about 60% is observed, correlated with two endothermic peaks at 295 and 368 °C. These two peaks can be associated to a stage of the decomposition step, corresponding to the oxidative decomposition of cellulose fibers. Relative to Whatman paper, although without any peaks present, a small mass loss is observed until 120 °C, corresponding to water evaporation. One endothermic peak at 336 °C is also detected, accompanied by one decomposition step, with a weight loss of approximately 80%. This peak is correlated to the thermal decomposition of cellulosic fibers.

So, by observing the results obtained, it is possible to ensure that the substrates can be heated up to 200 °C, without damage and without losing their properties (temperature at which the mass of the sample starts to decrease due to decomposition, indicating the maximum working temperature for this type of substrate).

The X-ray diffraction (XRD) results (see Figure 2c,d, for tracing and Whatman paper, respectively) show that both types of paper present the characteristic peaks of native cellulosic fibers: $\left(1\bar{1}0\right)$, (110), (200) and (004) at 2θ = 14.9°, 2θ = 16.6°, 2θ = 22.7° and 2θ = 35°, respectively; which are in accordance with that reported in the literature [39,41]. Due to the high intensity of peak (200) is possible to conclude that both types of paper are highly crystalline. No impurities or other crystallographic phases were detected.

The crystallinity index, based on the “Segal peak-height method” can be inferred by using the XRD data and calculating the ratio between intensity of the crystalline peak (I_002_ − I_AM_) and the total intensity of peak (002) (I_002_) [42,43]. It was estimated a crystallinity index of 78% and 89% for tracing and Whatman paper, respectively, indicating that Whatman paper have less amorphous fibres and more type 1 cellulose.

### 2.2. UV/Ozone ZnO Seed Layer Treatment

**Figure 3:**
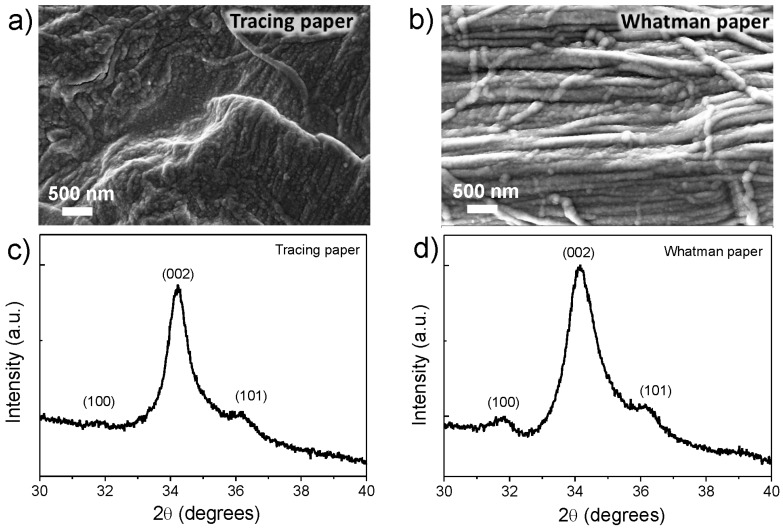
(**a**,**b**) SEM images and (**c**,**d**) XRD diffractograms of zinc oxide (ZnO) seed layer, deposited by sputtering technique on tracing and Whatman paper substrates, respectively.

After the deposition of ZnO seed layer on both cellulosic substrates, an UV/Ozone treatment was used in order to improve the ZnO surface polarity. In Figure 3 it is possible to observe the XRD measurement and SEM images of the ZnO seed layer, deposited by sputtering technique. It is possible to observe that the ZnO seed layer is very smooth with a preferable orientation along the (002) crystallographic plane.

The use of UV/Ozone systems for surface treatment have already been used by other authors in order to increase the surface oxygen, polarity and wettability [44,45]. This UV/Ozone system presents some advantages when compared with other systems: no vacuum is required (no need of any sophisticated apparatus), and the absence of a wet chemistry treatment gives the advantage of no residual solvents or other contaminants left at the substrate/sample surface; also, it can be used at room temperature [46].

**Figure 4:**
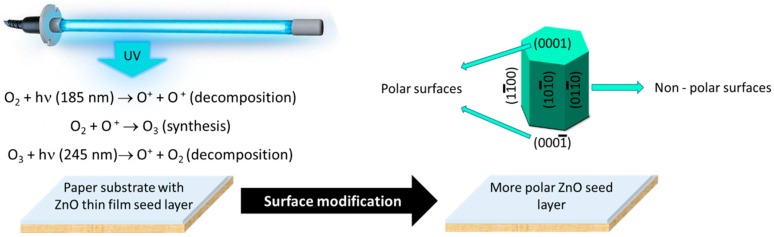
Schematic of surface treatment/modification of ZnO seed layer with an UV/Ozone system.

ZnO is a crystal with a hexagonal structure that grows along the *c* axis and possesses both polar and nonpolar surfaces, arising from the anisotropy of the wurtzite structure. It presents high energy polar surfaces, with a Zn^2+^ terminate (0001) plane and an O^2−^ terminated (000$\bar{1}$) plane (this surfaces reconstruct to lower the surface energy) [45,47]. As reported by Talebian et al., the solvents play an important role on hydrothermal/solvothermal synthesis [48]. When synthesizing ZnO nanoparticles by the hydrothermal method, it will originate a very strong interaction between the polar terminate plane (0001) and (000$\bar{1}$) of ZnO surfaces. When a ZnO nucleus is formed, due to the high energy of polar surfaces, the incoming precursor molecules will tend to adsorb on the polar surface of the ZnO seed layer. After adsorbing one layer of precursor molecules, the polar surface will transform into another polar surface, but with an inverted polarity (Zn^2+^ terminated surface will change into O^2−^ terminated surface, or vice versa). This process will be repeated over time, promoting the increase of the rate of crystal growth perpendicular to this surface (in the c-direction) and exposing the non-polar (1$\bar{1}00$) and (2$\bar{11}0$) surfaces [47,48,49]. So, by exposing the ZnO seed layer to UV light, the O_2_ adsorbed to the surface will be decomposed and the surface will become more polar, with a Zn terminate plane (0001), which will promote the growth of ZnO nanorods by hydrothermal synthesis method assisted by microwave irradiation. A more polar ZnO seed layer will improve the growth of ZnO nanorods with a direction perpendicular to the seed layer surface. Figure 4 shows a schematic of surface modification prior to ZnO nanorod arrays grown by hydrothermal method under microwave irradiation [50].

### 2.3. Crystallographic Structure and Morphology Analysis of ZnO Nanorods

**Figure 5:**
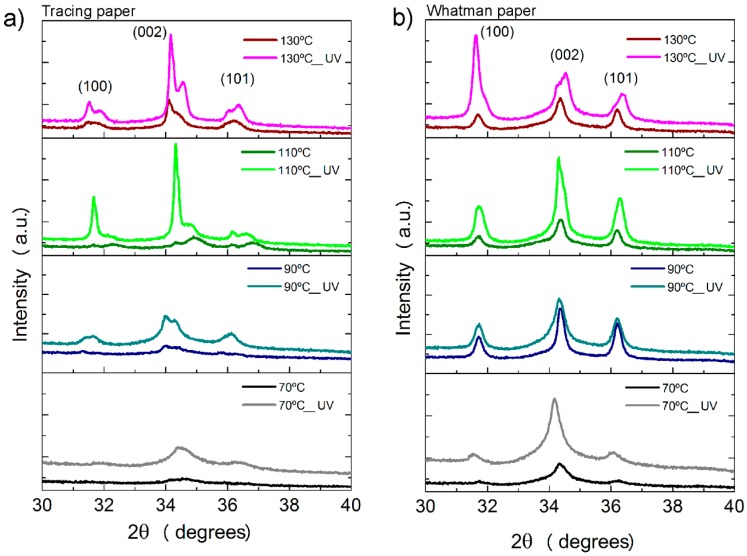
XRD diffractograms of ZnO nanorods arrays produced by an ultrafast hydrothermal method assisted by microwave irradiation, grown on (**a**) tracing paper substrate and (**b**) Whatman paper substrate. All samples were produced with a synthesis time of 10 min.

To infer the crystallographic structure and the morphology of the synthesized ZnO nanorod arrays, XRD and SEM analysis were carried out to all materials produced on this study. The XRD diffractograms of the ZnO nanorods grown on cellulosic substrates are presented in Figure 5. For all of the materials produced, the observed peaks can be fully indexed to the hexagonal wurtzite ZnO structure, with lattice constants of a = b = 0.3296 nm and c = 0.52065 nm, which is in accordance with the literature [51]. The three peaks observed are fully assigned to the crystallographic planes (100), (002) and (101). The results confirm that it was possible to grow pure ZnO nanocrystals on cellulosic substrates (tracing and Whatman papers). Nevertheless, is possible to observe that the crystallinity of ZnO nanorods increases with the increase of synthesis temperature and with UV/Ozone treatments prior to synthesis. These results corroborate the previous assumption that the UV/Ozone treatment favor the growth of ZnO nanorods on the surface of cellulosic substrates.

On the tracing paper condition, a splitting of the XRD peak corresponding to the crystallographic plane (002) is observed, and it might be due to the peak doublet of K-alpha 1 and K-alpha 2 [52].

**Figure 6:**
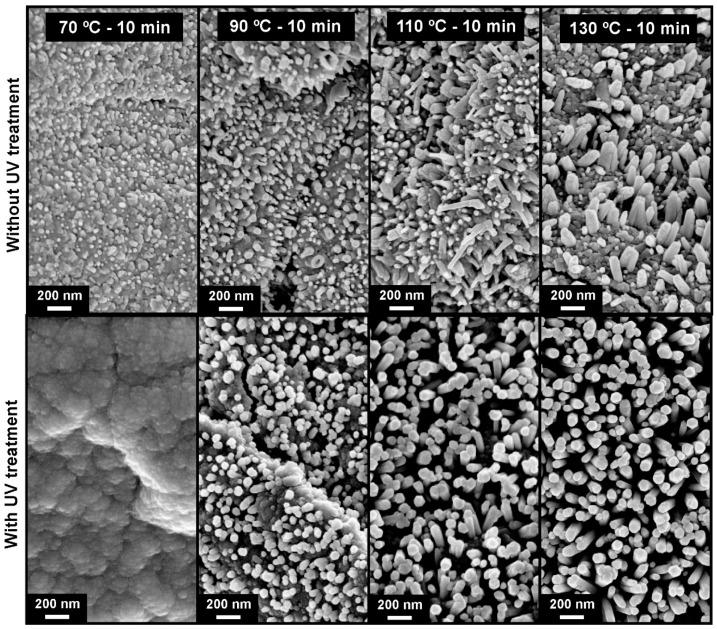
Surface SEM images of ZnO nanorods produced by hydrothermal method assisted by microwave irradiation on tracing paper, with a synthesis time of 10 min, with different synthesis temperatures (70, 90, 110 and 130 °C). On top are the images of samples produced without UV treatment and on the bottom samples produced with UV treatment.

SEM analysis for ZnO nanorod arrays synthesized on tracing paper substrate is shown on Figure 6. The effect of temperature and UV treatment prior to microwave synthesis has been investigated. The synthesis time was constant for all samples (10 min). It is possible to observe that without UV treatment, the ZnO nanorods grow in an inhomogeneous way, with a non-uniform shape and size (regardless the synthesis temperature). Moreover, it is easily observed that the ZnO nanorods do not cover fully the substrate surface. With a UV treatment prior to ZnO nanorod array synthesis, the growth of these nanostructures becomes more homogeneous, covering all the substrate surface. For low temperature synthesis (70 °C), no ZnO nanoparticle growth is observed, but with the increase of synthesis temperature the ZnO nanorods start to cover all of the substrate surface, becoming higher in length and well aligned, especially in the case of the tracing paper substrate, mainly due to its very smooth surface. Thus, with the increase of temperature, it is possible to observe that the nanorods become higher and thicker, for synthesis with UV treatment. The ZnO nanorods present an average length of 130 nm, 300 nm and 500 nm for synthesis temperatures of 90, 110 and 130 °C, respectively. Regarding the thickness, they present an average value of 85, 100 and 110 nm for the same range of temperatures.

**Figure 7:**
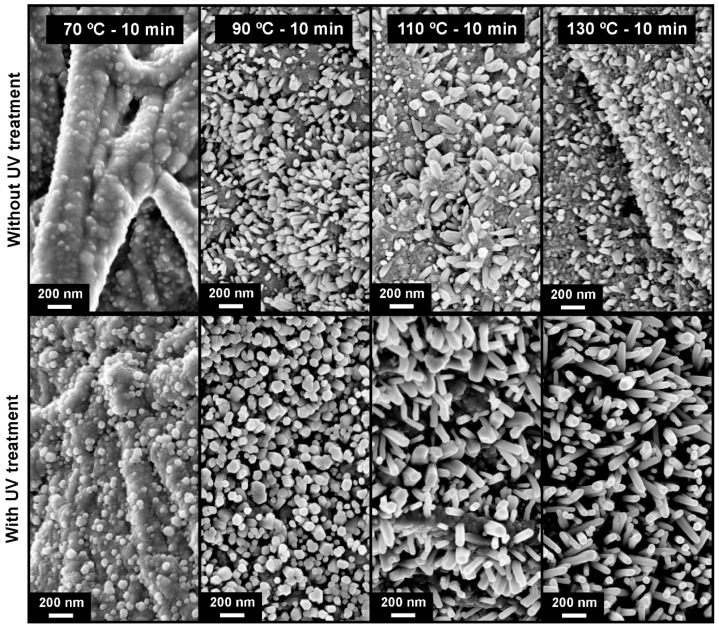
Surface SEM images of ZnO nanorods produced by hydrothermal method assisted by microwave irradiation on Whatman paper, with a synthesis time of 10 min, with different synthesis temperatures (70, 90, 110 and 130 °C). On top are the images of samples produced without UV treatment and on the bottom samples produced with UV treatment.

Figure 7 shows the SEM images of the synthesis of ZnO nanorod arrays using Whatman paper as substrate. Also in this case the growth as a function of synthesis temperature is observed, with and without an UV treatment prior to synthesis. As observed in tracing paper, it is possible to see that the use of an UV treatment prior to ZnO growth will favor the growth of this type of nanostructures. Without UV treatment, the growth is very heterogeneous, not completely covering the substrate surface. With UV treatment, the ZnO nanorod arrays grew uniformly, covering all of the surface. By increasing the synthesis temperature, the ZnO nanorods increased their length, presenting average values of 120, 340 and 480 nm for synthesis temperatures of 90, 100 and 130 °C, respectively. Regarding the thickness, they present an average value of 55, 66 and 75 nm for the same range of temperatures.

The misalignment of nanorods with the increase of temperature is due to the high roughness of the surface.

So, the top view SEM images (from both type of substrates) indicate that well-defined crystallographic planes of the hexagonal single crystalline ZnO nanorods can be identified and that they grow along the [0001] direction. Nevertheless, the growth in length is more expressive than in thickness, which implies that the growth rate must be along [0001] directions, and this latter direction is more temperature-sensitive when compared to those along [10$\bar{1}$1] and [10$\bar{1}$0] directions [53].

**Figure 8:**
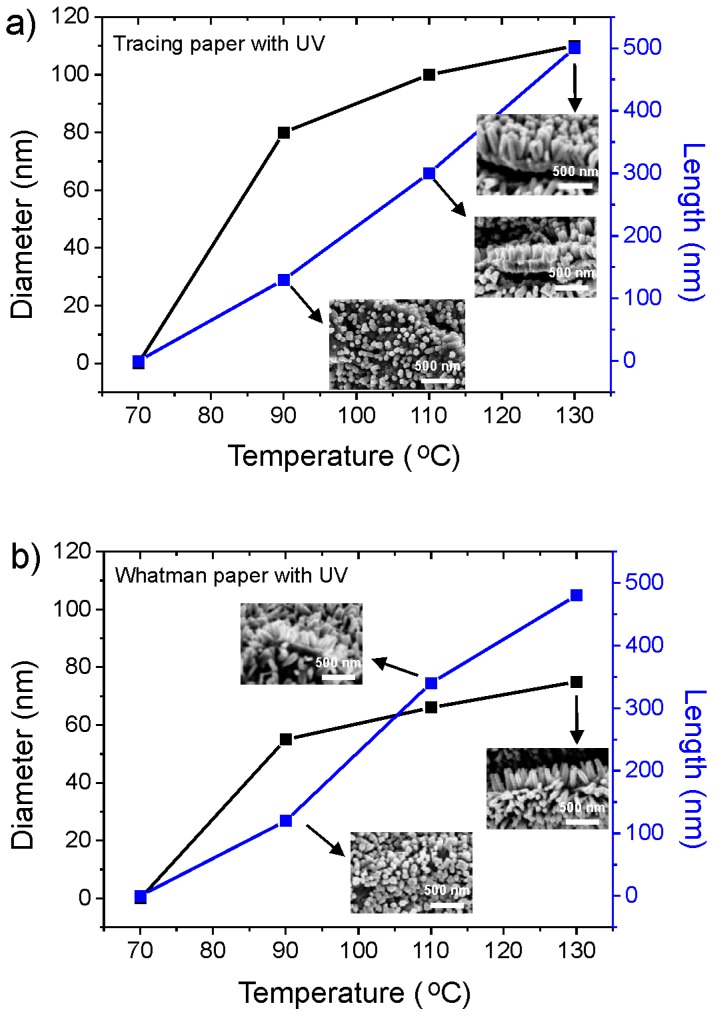
Behavior of length and diameter of the ZnO nanorods as a function of temperature for (**a**) tracing paper and (**b**) Whatman paper. The insets reveal the corresponding SEM images.

Comparing the two types of papers used, it is possible to conclude that tracing paper needs a higher temperature for ZnO nanorods start to nucleate at the surface. On Figure 8 is possible to observe a comparison between the length and the diameter of the ZnO nanorods as a function of temperature, for both type of cellulosic substrates, with the corresponding SEM images.

The small nanorods with and a relatively large length result in a high specific area, which is an important parameter in UV sensor application.

### 2.4. Optical Properties

The optical band gap of ZnO nanorods grown on cellulosic substrates was evaluated from reflectance results. It was applied the Tauc equation to reflectance values, for direct band semiconductors (see Equation (1)) [54]:

(1) $${\left(\alpha h\nu \right)}^{m}=A\left(h\nu -E_{g}\right)$$

where *E_g_* is the material optical band gap, *h* is the Plank constant (*h* = 4.135 × 10^−15^ eV s), *ν* is the frequency, *α* is the material absorption coefficient, m is a constant that depends on the type of the optical transition (*m* = 2 for allowed indirect transitions and *m* = 1/2 for allowed direct transition) and A is an energy-independent constant and.

**Figure 9:**
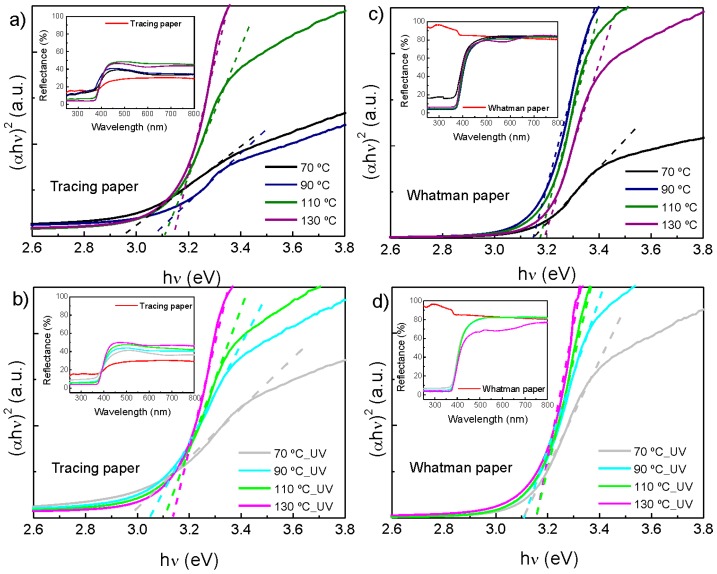
Absorbance spectra of ZnO nanorods arrays produced by an ultrafast hydrothermal method assisted by microwave irradiation, grown on (**a**,**b**) tracing paper substrate and (**c**,**d**) Whatman paper substrate, without and with UV/Ozone treatment. All samples were produced with a synthesis time of 10 min.

Figure 9 shows the optical band gap calculation for ZnO nanorods synthesis with and without UV treatment and as a function of synthesis temperature. The optical band gap was calculated by extrapolating (*αhν*)^2^ as a function of *hν*. On the insets, it is possible to observe the reflectance behavior of each sample, that is, the ZnO nanorod arrays produced absorb almost all the light in the UV region of the spectra.

The estimated optical band gap values, calculated from the Tauc equation, are discriminated on Table 1. By observing the values obtained it is possible to see that for both types of cellulosic substrates the band gap value increased with the increase of synthesis temperature, with or without the use of UV treatment prior to ZnO nanorods synthesis. This result is probably due to the increase of temperature, a more homogeneous nanorod arrays cover all the substrate surface, thus the measured band gap value become closer to the theoretical value ZnO band gap of 3.2–3.4 eV [55,56,57].

It is also possible to conclude that the same range of values was obtained, regardless of the type of cellulosic substrate used or if a UV treatment was used prior to ZnO nanorod synthesis.

### 2.5. Application of ZnO Nanorods in Paper-Based UV Sensors

The ZnO nanorods arrays grown on cellulosic substrates were applied as UV sensors, and for that only one condition from each substrate has been selected. By observing the SEM images (see Figure 6 and Figure 7) it was decided to test the ones with higher surface coverage and larger ZnO nanorods—Synthesis condition of 130 °C.

**Figure 10:**
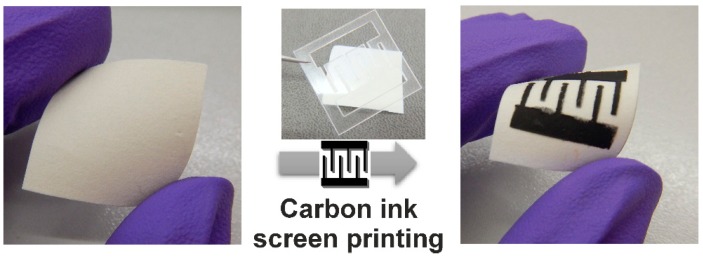
Schematic of the carbon interdigital contacts deposited by inkjet printing on cellulosic substrates.

So, for ZnO nanorod UV sensor production, carbon interdigital contacts were screen printed on paper substrates after ZnO nanorods growth, like is exemplified in Figure 10. A polymeric mask was used with the interdigital contacts design that allow us to ensure that the contacts were equal for both the samples.

The samples were then subjected to UV radiation cycles, with a bias voltage of +10 V and on/off cycles of 2 min.

The sensing mechanism is based on the oxygen vacancies that exist on the surface and that will influence the properties of ZnO nanoparticles. In the dark, molecules containing high concentrations of O_2_ are adsorbed at vacancy sites that accept electrons, which will be withdrawn and effectively depleted from the conduction band. This mechanism will lead to a conductivity reduction. When exposed to UV light, reducing molecules will react with the adsorbed oxygen, leaving behind an electron, inducing an increase in the electrical conductivity [19,58,59]. This phenomena can be described by the following equations [60]:

(2) $$O_{2}\left(g\right)+e^{-}\;\to \;O_{2}^{-}\;\left(ad\right)$$

(3) $$h\nu \;\to \;e^{-}+h^{+}$$

(4) $$h^{+}+O_{2}^{-}\left(ad\right)\to O_{2}\left(g\right)$$

**Figure 11:**
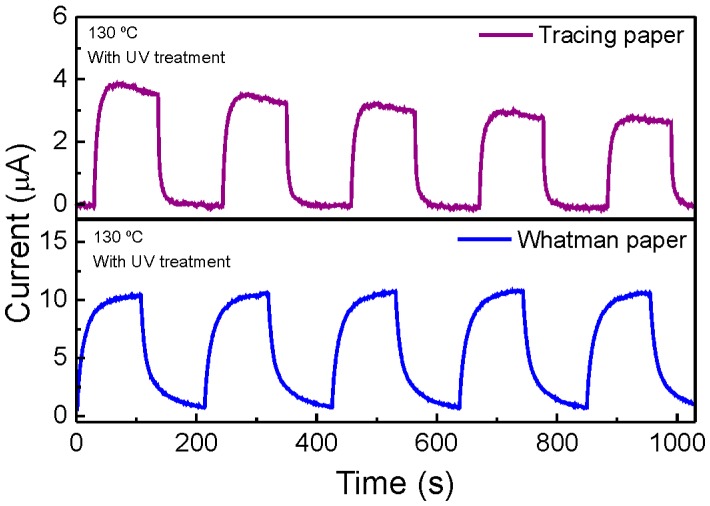
Cycling behavior of cellulosic ZnO nanorods UV sensors grown on tracing paper and on Whatman paper.

In Figure 11, it is possible to observe the time resolved photocurrent of ZnO nanorods paper UV sensor, produced for 10 min and 130 °C on tracing and Whatman substrates, in response to UV radiation turn on/off. The ZnO synthesized on Whatman paper substrates displayed enhanced sensing performance when compared to tracing paper substrate. Under the bias voltage of +10 V, the photocurrent exponential increase from 16.6 nA to 3.8 µA for tracing paper while for Whatman paper increased from 0.76 µA to 10.36 µA, within about 60 s, with saturation in the on-state. After the UV radiation was turned off, the current decreased to the initial value stage of current. The photocurrent of ZnO UV sensors was completely reproducible during several cycles of photocurrent switching. This behavior was obtained for both types of paper. The low values of photocurrent observed in both UV sensors may be related to the use of paper as a substrate. They are rough and present a high porosity. Moreover, after ZnO nanorods synthesis the tracing paper becomes slightly wrinkled, which may make its use as an UV sensor device difficult. These characteristics will make the current flow through the sample difficult. Regarding the nanorods’ influence, no significant structural differences (in length and diameter) have been observed between the nanorod arrays produced in both paper substrates, confirming that the major contribution must come from the substrate.

In order to determine the responsivity, *R*, of each UV ZnO paper sensor it was used the following equation [61]:

(5) $$R=\frac{I_{ph}-I_{dark}}{P_{UV}}$$

where *P_UV_* is the power of the UV source lamps, *I_ph_* is the UV sensor photocurrent and *I_dark_* is the UV sensor dark current. The obtained responsivity was 0.39 µA/W and 1.19 µA/W, for the ZnO nanorods on tracing paper and on Whatman paper, respectively. The responsivity was calculated taking into account the current value when the sensor reaches 95% of its stable value [62].

So, the ZnO nanorods UV sensor produced on Whatman substrate presents a photo response 3 times superior to the one produced in tracing substrate. It is expected to occur due the grain size effect. It is well known that the sensitivity of a nanostructured sensor is related to grain size, with the particle geometry, oxygen absorption and lattice defects. Smaller grain size will induce a higher sensor sensitivity due to an increase of the specific surface area and oxygen adsorption quantity [63]. In order to infer the grain size of the ZnO nanorods grown on tracing and Whatman paper substrates, it was used the Scherres’s equation, D = 0.94 λ/β cosθ, where λ is the X-ray radiation wavelength, θ is Bragg’s angle β is the full width at half maximum [64]. A grain size of 69 nm and 26 nm was estimated for ZnO nanorods grown on tracing and Whatman paper, respectively, which may justify the higher value of responsivity obtained with UV sensor on Whatman substrate.

**Figure 12:**
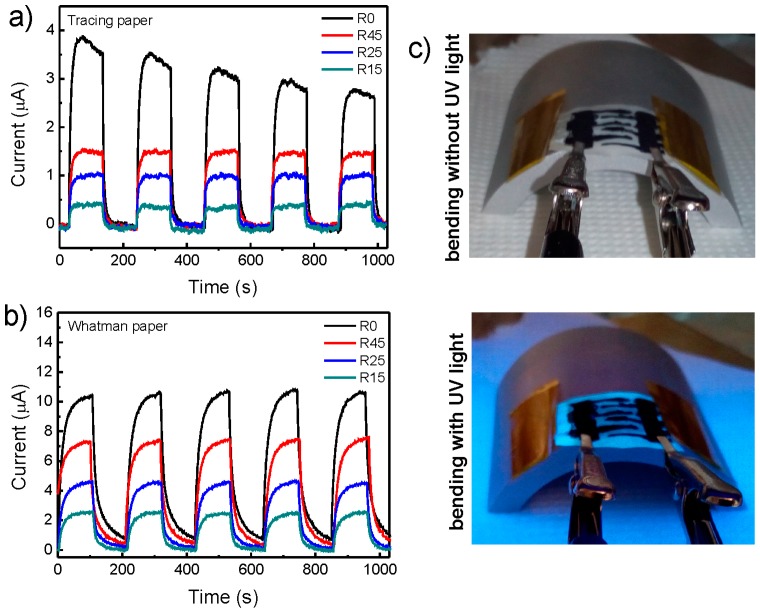
Flexibility cycling behavior of cellulosic ZnO nanorods UV sensors grown on (**a**) tracing paper and (**b**) Whatman paper as a function of a curvature radius of 45, 25 and 15 cm (R45, R25 and R15, respectively). R0 is the measurement with no bending. (**c**) Photograph of cellulosic ZnO nanorods UV sensor, in a bending state, without and with UV light.

The flexibility of the UV sensors produced was also tested, by placing them on round mods with curvature radius of 45, 25 and 15 cm. The results are shown on Figure 12. Both sensors produced on tracing and Whatman paper substrates show a decrease in the responsivity for smaller bending radius and consequently higher strains. This can be explained both by the device resistance increment, and by alteration of the light interaction with the sensor, which may be less efficient for larger angular scattering. When strain is induced to the ZnO nanorod arrays, it will induce the formation of piezoelectric polarizations charges that can promote the oxygen adsorption/re-adsorption processes, reducing the sensor responsivity [65].

In Table 2, it is possible to observe the measured parameters, as a function of the curvature radius, of ZnO nanorods UV sensors produced on tracing and Whatman papers. It is also possible to observe that the response time and the recovery time does not change much with the curvature radius, presenting a higher value for Whatman paper that may be due to the high porosity observed for this type of substrate.

## 3. Experimental Details

### 3.1. Synthesis of ZnO Nanostructures

An ultrafast method based on hydrothermal synthesis assisted by microwave irradiation have been used for the synthesis of ZnO nanorod arrays on paper substrate. Two distinct types of paper substrates coated with a ZnO seed thin film layer have been used: tracing (Canson, Annonay, France) and Whatman (Sigma-Aldrich, St. Louis, MO, USA) no. 2 papers.

The ZnO seed layer was deposited on the two types of paper substrates by radio frequency (RF) sputtering, at room temperature. A ceramic oxide target of ZnO with a purity of 99.99% was used for the deposition. For the depositions, the chamber was evacuated to a base pressure of 10^−6^ mbar. A shutter between the substrate and the target enabled the protection of the targets from cross contamination. For the deposition of ZnO seed layer, it was used a power density of 12.30 Wcm^−2^ and a deposition pressure of 4 × 10^−3^ mbar. The distance between the target and substrate was fixed at 15 cm. The deposition was carried out for 90 min allowing the formation of a 200 nm ZnO layer.

After uniformly coating the two types of paper substrates with ZnO thin films, ZnO nanorod arrays were grown by hydrothermal synthesis under microwave irradiation with a Discover SP microwave system, from CEM (Matthews, NC, USA). Two different approaches were tested, UV/ozone treated or without any previous treatment. For UV treatment, the substrates (tracing and Whatman no. 2) were placed for 5 min in a UV/Ozone system from Novascan (Bonne, MO, USA), equipped with two UV lamps with wavelengths of 185 nm and 254 nm. The distance between the paper and UV lamps were kept at 10 cm. For the microwave-assisted synthesis, the ZnO seeded substrates (20 × 20 mm) were placed at an angle against the Pyrex vessel, with the seed layer facing down [16] and filled with an aqueous solution of 25 mM zinc nitrate hexahydrate (Zn(NO_3_)_2_·6H_2_O; 98%, CAS: 10196-18-6) and 25 mM hexamethylenetetramine ((C_6_H_12_N_4_)_2_; 99%, CAS: 100-97-0) [12], both from Sigma Aldrich (St. Louis, MO, USA). Microwave synthesis was fixed at 10 min.

**Figure 13:**
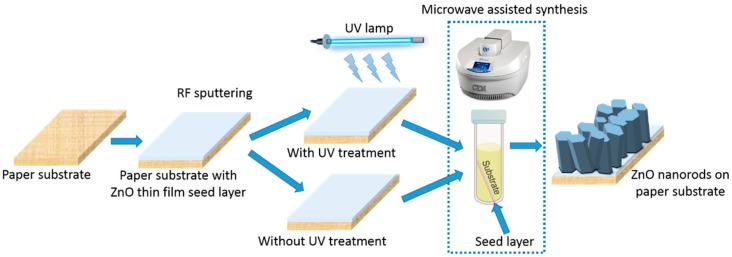
Schematic of ZnO nanorods hydrothermal synthesis assisted by microwave irradiation on paper substrate with a ZnO thin film seed layer deposited by sputtering method.

The use of a UV/Ozone treatment was tested before the nanorods synthesis and was optimized the growth temperature to obtain a uniformly coated paper with ZnO nanorods. The synthesis was done at different temperatures: 70, 90, 110 and 130 °C. After each synthesis process, the paper substrates were cleaned with deionized water and ethanol and dried with nitrogen compressed air. Figure 13 represents a schematic of the production process for ZnO nanorods arrays synthesis on paper substrates (with and without an UV/Ozone treatment).

### 3.2. Characterization Techniques

Differential scanning calorimetric (DSC) measurements of tracing and Whatman paper substrates were carried out using a Simultaneous Thermal Analyser (TGA-DSC-STA 449 F3 Jupiter, Netzsch-Geratebau GmnH, Selb, Germany). Approximately 5–7 mg of each sample was loaded into an open aluminium crucible and heated from room temperature to 550 °C with a heating rate of 5 °C min^−1^, in air atmosphere.

Surface 3D profilometry of the paper substrate was performed using an Ambios XP-200 (Ambios Technology, Santa Cruz, CA, USA) profiler for an area of 1 × 1 μm^2^.

The crystallinity of the ZnO nanorod arrays has been determined X-ray diffraction (XRD), using a PANalytical’s X’Pert PRO MRD X-ray diffractometer, (PANalytical B.V., Almero, The Netherlands) with a monochromatic CuKα radiation source (wavelength 1.540598 Å). XRD measurements have been carried out from 10° to 90° (2θ), for paper analysis, and from 30° to 40°, for ZnO nanorods measurements, with a scanning step size of 0.016°. The morphology of papers substrates and ZnO nanorods has been characterized by Scanning Electron Microscopy (SEM) using a Carl Zeiss AURIGA CrossBeam Workstation instrument (Carl Zeiss Microscopy GmbH, Oberkochen, Germany) equipped with an Oxford X-ray Energy Dispersive Spectrometer (Carl Zeiss Microscopy GmbH, Oberkochen, Germany). The length and width of the ZnO nanorods were determined from SEM micrographs using 20 individual nanorods, using ImageJ software [66].

Room temperature diffuse reflectance measurements of were performed using a Perkin Elmer lambda 950 UV/VIS/NIR spectrophotometer (Perkin Elmer, Inc., Waltham, MA, USA) with a diffuse reflectance module (150 mm diameter integrating sphere, internally coated with Spectralon). The calibration of the system was achieved by using a standard reflector sample (reflectance, R = 1.00 from Spectralon disk). The reflectance (R) was obtained from 250 to 800 nm.

### 3.3. Characterization of ZnO Nanorods on Tracing and Whatman Substrate as a UV Sensor

The synthesized ZnO nanorod arrays on tracing and Whatman paper substrates were characterized as a UV sensor, using a potentiostat model 600, from Gramy Instruments, Inc. (Warminster, PA, USA), in a chronoamperiometry configuration, with a constant applied voltage of 10 V. For interdigital electrical contacts, a carbon resistive ink, PE-C-774, from Conductive Compounds (Hudson, NH, USA), was used. The ZnO nanorod arrays were subjected to UV irradiation with an EL-Series Twin Tube UV lamps, from UVP (Upland, CA, USA) with an intensity of 8 W at a wavelength of 365 nm. The sensor produced was irradiated for 2 min, followed by 2 min in the off state.

## 4. Conclusions

In the present work, the synthesis of ZnO nanorod arrays was studied on two different cellulosic substrates, tracing and Whatman papers. An ultra-fast chemical synthesis method, based on microwave irradiation, was employed requiring just 10 min to produce ZnO nanorod arrays in both substrates. The influence of a UV treatment prior to synthesis was studies together with the synthesis temperature effect on the growth of ZnO nanorod arrays on paper substrates. It was observed that without UV treatment, the growth on ZnO was heterogeneous, not covering all the substrate surface. With the use of UV treatment and increase of synthesis temperature, larger ZnO nanorods fully covering both cellulosic substrates could be observed. The XRD analysis confirmed the formation of pure and crystalline wurtzite ZnO, with no other impurities detected. The samples produced at 130 °C, with UV treatment prior to ZnO synthesis, were characterized as a UV sensor, revealing an increase of 3 times in the responsivity with the use Whatman paper, when compared with tracing paper. Bending tests revealed that decreasing the curvature radius will also decrease the responsivity of the UV sensor. Nevertheless, the results show that these types of sensor are stable when working in a bending mode.

## Figures and Tables

**Table 1:** Optical band gap of ZnO nanorods, produced with different synthesis time and temperature, obtained by extrapolating (*αhν*)^2^ vs. *hν*.

Synthesis Temperature		70 °C	90 °C	110 °C	130 °C
Tracing paper	Without UV treatment	2.95 eV	3.07 eV	3.10 eV	3.14 eV
	With UV treatment	2.96 eV	3.05 eV	3.11 eV	3.14 eV
Whatman paper	Without UV treatment	3.15 eV	3.15 eV	3.18 eV	3.19 eV
	With UV treatment	3.10 eV	3.10 eV	3.16 eV	3.16 eV

**Table 2:** UV Sensor parameters measured as a function of a curvature radius of 45, 25 and 15 cm.

Paper	Radius	Response Time (s)	Recovery Time (s)	Responsivity (µA/W)
Tracing	R0	30	27	0.39
	R45	27	25	0.19
	R25	24	26	0.12
	R15	21	30	0.044
Whatman	R0	57	65	1.20
	R45	57	62	0.84
	R25	62	57	0.57
	R15	61	48	0.32
